# Supplementary material for: Heterozygous inversion breakpoints suppress meiotic crossovers by altering recombination repair outcomes
Source: PLoS Genet. 2023 Apr 13;19(4):e1010702. doi: 10.1371/journal.pgen.1010702 (PMC10128924; doi:10.1371/journal.pgen.1010702)
Supplement: S1 Fig — PCR results show that primer sets fail to amplify a specific band in Oregon-RM, but do amplify specific bands of the expected sizes in dl-49, confirming that the expected breakpoints are present. (DOCX) [file pgen.1010702.s001.docx]

| Primer  Set | Breakpoint | Forward primer | Primer sequence | Reverse primer | Primer sequence | Expected size (bp) |
| --- | --- | --- | --- | --- | --- | --- |
| A | Proximal | dl49proximal_for1 | CGCCTGCCTACAACAAAGTT | dl49proximal_rev1 | TTCCTTCGGATGCTTGGACT | 605 |
| B | Proximal | dl49proximal_for2 | TGGTGGAAAATCGGATCGGA | dl49proximal_rev2 | CCATAATTAACGTGCCGCCA | 647 |
| C | Proximal | dl49proximal_for3 | CACACGCTCACATGTCACAT | dl49proximal_rev3 | GTTGGAAAGGACACGACGAC | 734 |
|  |  |  |  |  |  |  |
| D | Distal | dl49distal_for1 | CAGCAACATCAGTACAGCGG | dl49distal_rev1 | CACAGATCTACAGCCACCGA | 942 |
| E | Distal | dl49distal_for2 | CGAATCTCAGCCGCTTTCTC | dl49distal_rev2 | ATGCGGGTCTGGAATGTAGG | 983 |
| F | Distal | dl49distal_for3 | TCCTCCTTCGAATCTCAGCC | dl49distal_rev3 | GATTTGCACGCTGGTAAACC | 544 |


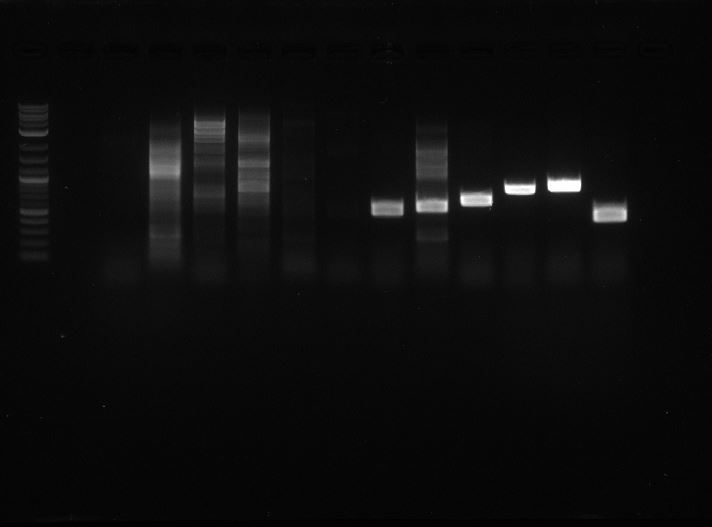


Ladder

A B C D E F

A B C D E F

Oregon-RM

*dl-49*

## Supplemental Figure 1. PCR primers used to detect the dl-49 breakpoints. PCR results show that primer sets fail to amplify a specific band in Oregon-RM, but do amplify specific bands of the expected sizes in dl-49, confirming that the expected breakpoints are present.
