# Supplementary material for: Heterozygous inversion breakpoints suppress meiotic crossovers by altering recombination repair outcomes
Source: PLoS Genet. 2023 Apr 13;19(4):e1010702. doi: 10.1371/journal.pgen.1010702 (PMC10128924; doi:10.1371/journal.pgen.1010702)
Supplement: S2 Fig — A) We previously isogenized dl-49 and crossed in an isogenized chr2 from Oregon-RM. This stock was used for cross 1 in Fig 2. The next several generations were needed to create a stock that was heterozygous for dl-49 and chrX from Oregon-RM and that shared genetic background on chr3. Full-sibling stocks were created at generation 10 by crossing dl-49 heterozygotes to either dl-49 males (B) or Oregon-RM males (C). This cross scheme did not result in isogenized 3rd chromosome but did create a shared genetic background. (DOCX) [file pgen.1010702.s002.docx]

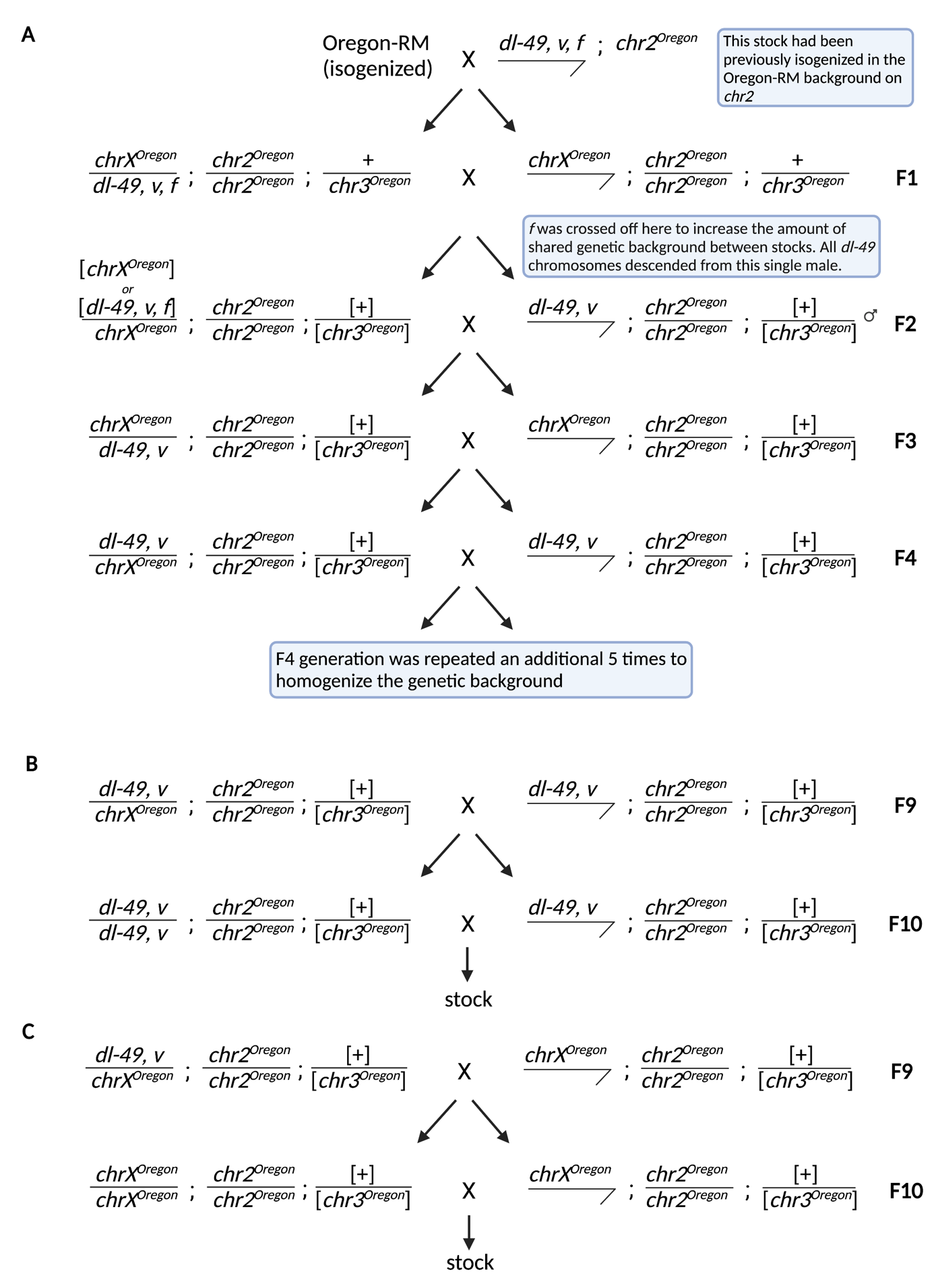


Supplemental Figure 2. Crosses used to generate full sibling Oregon-RM and *dl-49* stocks. A) We previously isogenized *dl-49* and crossed in an isogenized *chr2* from Oregon-RM. This stock was used for cross 1 in Figure 2. The next several generations were needed to create a stock that was heterozygous for *dl-49* and *chrX* from Oregon-RM and that shared genetic background on *chr3*. Full sibling stocks were created at generation 10 by crossing *dl-49* heterozygotes to either *dl-49* males (B) or Oregon-RM males (C). This cross scheme did not result in isogenized 3^rd^ chromosome but did create a shared genetic background.
