# Supplementary material for: Heterozygous inversion breakpoints suppress meiotic crossovers by altering recombination repair outcomes
Source: PLoS Genet. 2023 Apr 13;19(4):e1010702. doi: 10.1371/journal.pgen.1010702 (PMC10128924; doi:10.1371/journal.pgen.1010702)
Supplement: S3 Fig — Top panel is number of SNVs between Oregon-RM and y cv wy f; bottom panel is number of SNVs between dl-49 and y cv wy f. The extremely poor SNV density between dl-49 and y cv wy f on the distal end can be seen. (DOCX) [file pgen.1010702.s003.docx]

*Supplemental Figure 3.* Number of SNVs per 50 kb windows with 25 kb overlap between windows. Top panel is number of SNVs between Oregon-RM and y cv wy f; bottom panel is number of SNVs between dl-49 and y cv wy f. The extremely poor SNV density between dl-49 and y cv wy f on the distal end can be seen.
