# Supplementary material for: Heterozygous inversion breakpoints suppress meiotic crossovers by altering recombination repair outcomes
Source: PLoS Genet. 2023 Apr 13;19(4):e1010702. doi: 10.1371/journal.pgen.1010702 (PMC10128924; doi:10.1371/journal.pgen.1010702)
Supplement: S4 Fig — These distributions are not significantly different from each other and were combined into one dataset (Kolmogorov-Smirnov test, p = 0.61). (DOCX) [file pgen.1010702.s004.docx]

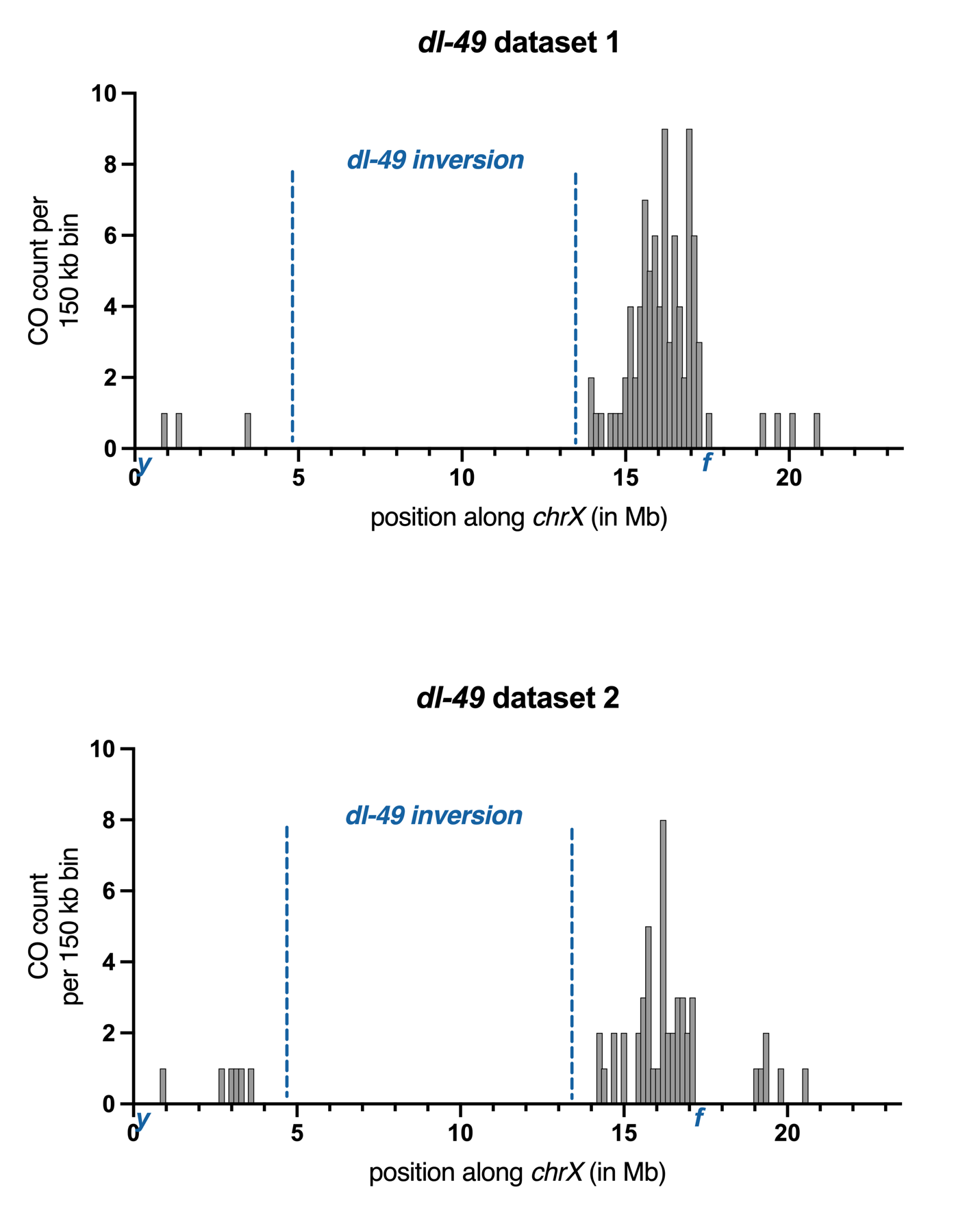


## Supplemental Figure 4. CO frequencies in dl-49 heterozygotes from cross 1 and cross 2 (Figure 2). These distributions are not significantly different from each other and were combined into one dataset (Kolmogorov-Smirnov test, p = 0.61).
