# Supplementary material for: Heterozygous inversion breakpoints suppress meiotic crossovers by altering recombination repair outcomes
Source: PLoS Genet. 2023 Apr 13;19(4):e1010702. doi: 10.1371/journal.pgen.1010702 (PMC10128924; doi:10.1371/journal.pgen.1010702)
Supplement: S5 Fig — A) Raw counts of CO frequencies in Oregon-RM. 96 COs between y and cv or wy and f were sequenced. COs that occur between cv and wy were from samples that had more than one CO on the chromosome. These COs were not included in any analysis. B) Raw counts of CO frequencies from dl-49 heterozygotes. 145 COs between y and f were sequenced. (DOCX) [file pgen.1010702.s005.docx]

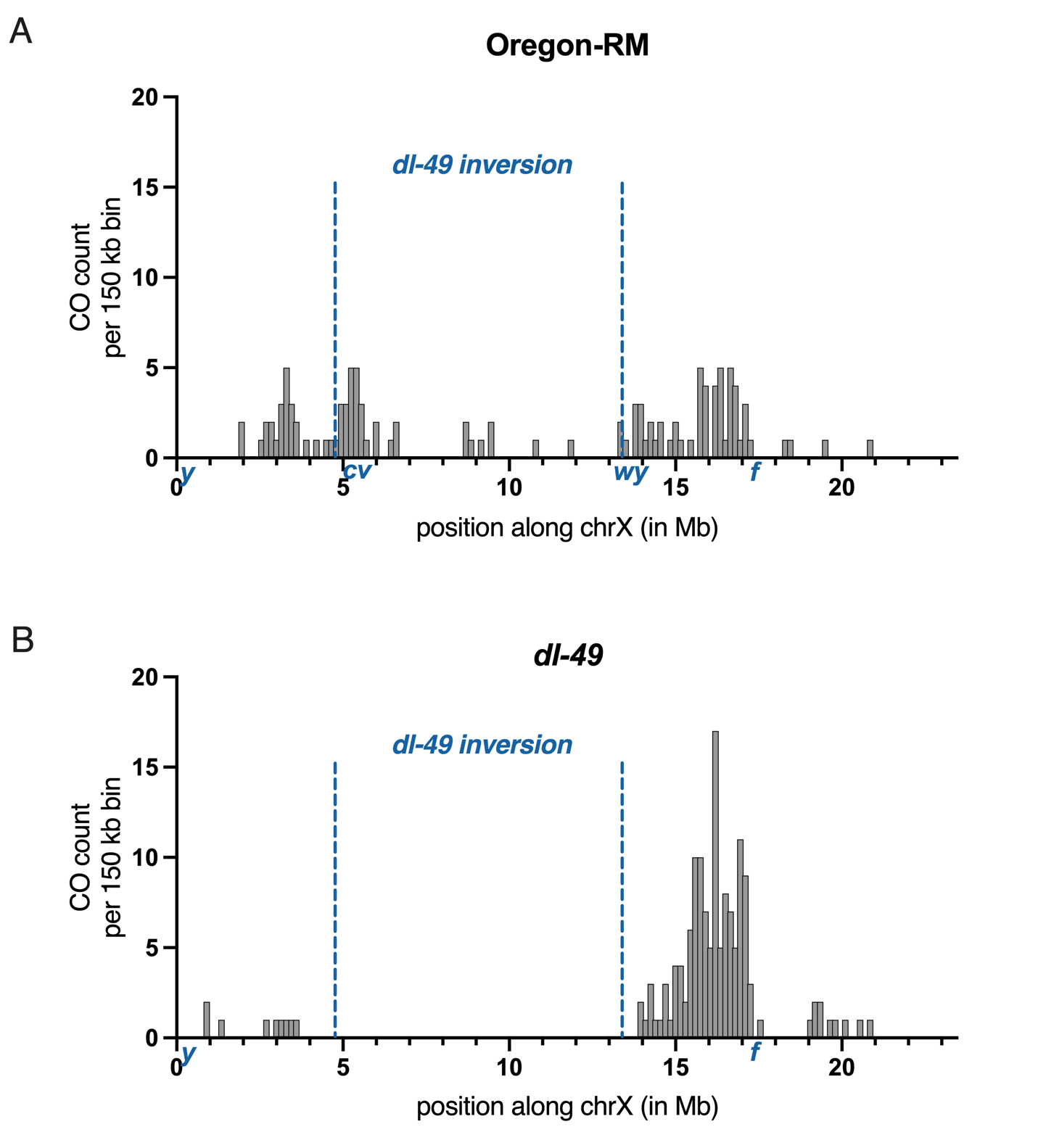


## Supplemental Figure 5. A) Raw counts of CO frequencies in Oregon-RM. 96 COs between y and cv or wy and f were sequenced. COs that occur between cv and wy were from samples that had more than one CO on the chromosome. These COs were not included in any analysis. B) Raw counts of CO frequencies from dl-49 heterozygotes. 145 COs between y and f were sequenced.
