# Supplementary material for: Heterozygous inversion breakpoints suppress meiotic crossovers by altering recombination repair outcomes
Source: PLoS Genet. 2023 Apr 13;19(4):e1010702. doi: 10.1371/journal.pgen.1010702 (PMC10128924; doi:10.1371/journal.pgen.1010702)
Supplement: S6 Fig — Table lists the location of NCOGC, primers used for PCR and sequencing, expected PCR product size, and the samples sequenced. Sanger sequencing traces are shown for each sample and are in the same order as in the table. (DOCX) [file pgen.1010702.s006.docx]

| Coordinates of NCOGC | Primer name | Primer sequence | Primer name | Primer sequence | Expected PCR product size | Samples sequenced |
| --- | --- | --- | --- | --- | --- | --- |
| chrX:13042511-13042524 | dl49_val2_for1 | TCTCGGCTATGCAACTCAAA | dl49_val2_rev1 | CCCCAGCAGCTTATGAAATCA | 214 bp | *dl-49*_parent |
|  |  |  |  |  |  | *y cv wy f*_parent |
|  |  |  |  |  |  | dl-49_nonrec_51_S51 |
|  |  |  |  |  |  | dl-49_nonrec_61_S61 |
|  |  |  |  |  |  | dl-49_nonrec_77_S77 |
|  |  |  |  |  |  | dl-49_nonrec_48_S48 |
|  |  |  |  |  |  | dl-49_nonrec_20_S20 |
|  |  |  |  |  |  | dl-49_nonrec_13_S13 |
|  |  |  |  |  |  | dl-49_nonrec_12_S12 |
| chrX:9565406-9565406 | dl49_val3_for1 | TTGGAGATGGCGGAGTGTTT | dl49_val3_rev1 | CGAGACGGAAGATGACAAGC | 201 bp | *dl-49*_parent |
|  |  |  |  |  |  | *y cv wy f*_parent |
|  |  |  |  |  |  | dl-49_nonrec_95_S95 |
|  |  |  |  |  |  | dl-49_nonrec_46_S46 |
|  |  |  |  |  |  | dl-49_nonrec_36_S36 |
|  |  |  |  |  |  | dl-49_nonrec_16_S16 |


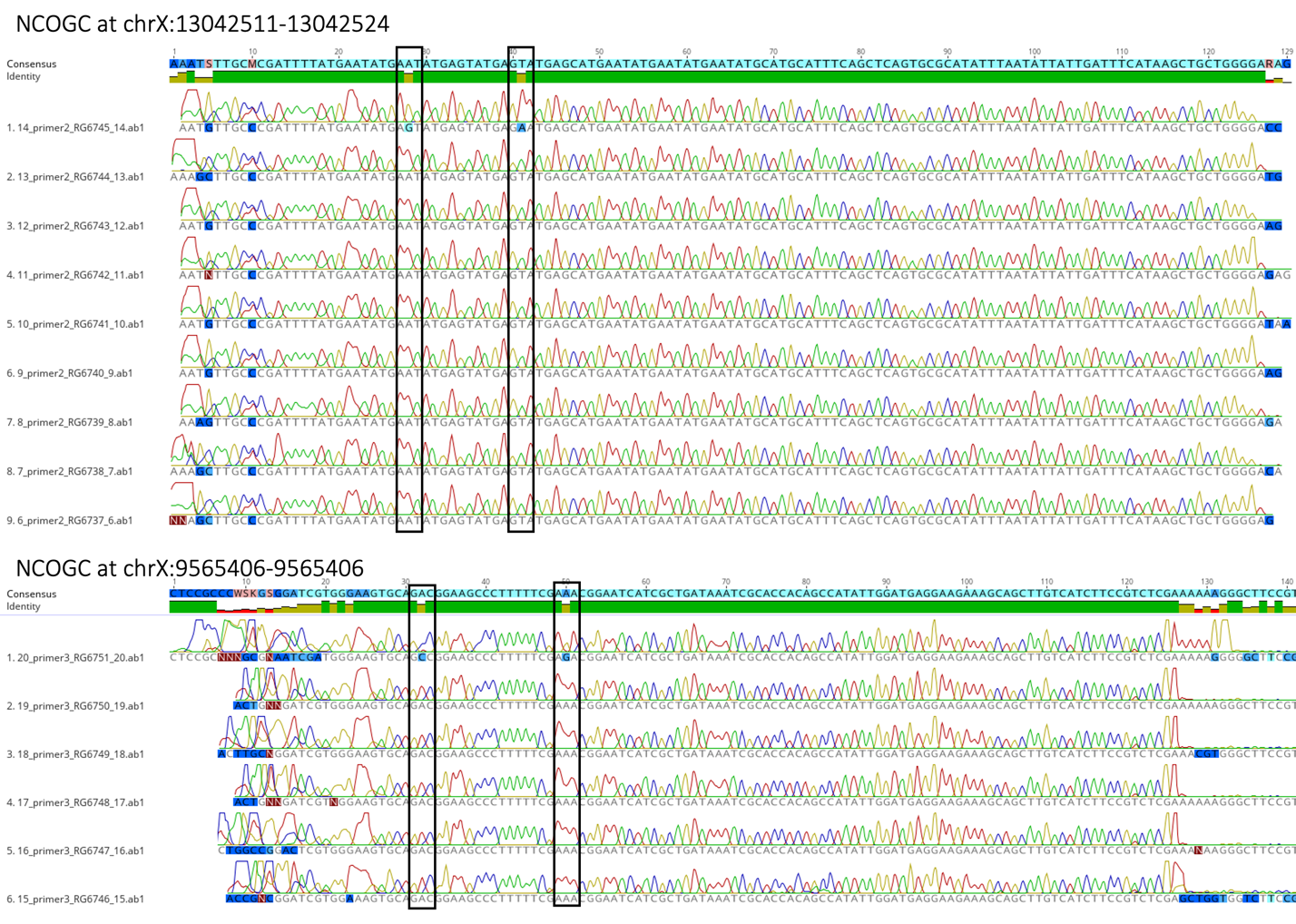


## Supplemental Figure 6. Sequencing confirmation of pre-meiotic NCOGC events. Table lists the location of NCOGC, primers used for PCR and sequencing, expected PCR product size, and the samples sequenced. Sanger sequencing traces are shown for each sample and are in the same order as in the table.
